# Enlarging the divide? Per-Capita Income as a measure of social inequalities in a southern European City

**DOI:** 10.1007/s11135-022-01360-6

**Published:** 2022-03-17

**Authors:** Kostas Rontos, Barbara Ermini, Luca Salvati

**Affiliations:** 1 Department of Sociology, University of the Aegean, University Hill, EL-81100 Mytilene, Greece; 2 Department of Economics and Social Science, Polytechnic University of Marche, Piazzale Martelli, 8, I-60121 Ancona, Italy; 3 Department of Economics and Law, University of Macerata, Via Armaroli 43, I-62100 Macerata, Italy

**Keywords:** Socio-spatial structure, Urban gradient, Regional disparities, Athens, Mediterranean cities

## Abstract

Earlier studies relating form and functions of cities address an intriguing and complex research issue, especially for specific urban typologies. Although with inherent differences on a local scale, Mediterranean cities represent diversified settlement morphologies and multifaceted socioeconomic contexts. The present study investigates the socioeconomic structure at the base of rapid development of a large Mediterranean agglomeration (Athens, Greece). Results of a multivariate analysis of the spatial distribution of average (per-capita) declared income and non-parametric correlations of contextual indicators suggest that the characteristic socio-spatial structure of the 1970s and the 1980s in Athens had slightly changed in recent times. A remarkable segregation in wealthy and disadvantaged communities - well beyond the urban-rural divide usually observed in Mediterranean regions - consolidated in recent decades. Despite economic transformations shaping urban design and infrastructural networks, persistent disparities between affluent and economically depressed neighborhoods still characterize the socio-spatial structure of contemporary Athens.

## Introduction

Factors leveraging metropolitan growth and change are intrinsically associated with socioeconomic conditions, making the analysis of urban form and functions a very articulated and inherently multidimensional issue (Crooks et al. 2015; Salvati and Serra 2016; Cuadrado-Ciuraneta et al., 2017). Structural changes consequent to recessions and, more recently, the new ‘revolution’ in the realm of digitalization induced by Covid-19 pandemic, have exerted significant impacts in the spatial organization of the fundamental functions of production and consumption (Brakman et al. 2015; De Rosa and Salvati 2016; Melguizo and Royuela 2020; Shutters et al. 2021).

By re-defining extensively the use of spaces at both local and regional scale, the pervasiveness of global economic networks leveraged a continuous process of spatial dispersion of resident population and workforce (Chung et al. 2020; Mbambo and Agbola 2020; Hesse and Rafferty 2020; Tranos and Ioannides 2021). Local firms and suppliers, and therein cities and regions, find opportunities to get access to larger markets and to new technologies, if they succeed to be part of a global value chain (Salvati et al. 2013; Chelleri et al. 2015; Perrin et al. 2018). Being part of such chains may also affect city size and metropolitan hierarchies across countries and continents (e.g. Malheiros and Vala 2004).

A heterogeneous distribution of jobs between central cities and suburbs contributed to alter both configuration and functions of urban spaces (Salvati et al. 2016; Di Feliciantonio et al. 2018; Barbarossa 2020). At the same time, intense social transformations were observed along urban gradients (Carlucci et al. 2018), since different functions are spatially intertwined and co-exist in a dynamic mixture of activities, productions, and services within metropolitan regions (Tao et al. 2020). Based on those dynamics, urban areas became progressively more dispersed and fragmented, with layers of overlapping functions and spatial co-occurrence of economic activities (Gerocs and Pinkasz 2019; Zeng 2019; Chen et al. 2020).

Identifying the main factors at the base of urban growth and change is a challenging research issue. Economic dynamics, the evolving socio-spatial structure, territorial patterns, cultural and political indicators, are identified as candidate drivers of metropolitan transformation (Ustaoglu and Williams 2017; Frick and Rodríguez-Pose 2018; Barrado-Timón et al. 2020; Wang et al. 2021). The synergic impact of the abovementioned processes is more evident in specific world regions (Turok and Mykhnenko 2007; Garcia-Nieto et al. 2018; Geddes 2020). In these regards, cities in Mediterranean Europe exhibit multifaceted development trajectories (Gospodini 2009; Diaz-Palacios-Sisternes et al. 2014; Cuadrado-Ciuraneta and Durà-Guimerà 2018), leading to a mostly heterogeneous spatial evolution of settlements, reflecting a broad spectrum of socioeconomic processes and metropolitan transformations (Vannier et al. 2019; Basile and Cavallo 2020; Kamalipour and Iranmanesh 2021).

Assuming Mediterranean as “one thousand things together - Not one, but innumerable landscapes” (Braudel 1987), a comprehensive interpretation of urban transformations in such a context is a hard task, because of the social, economic, institutional, and cultural uniqueness at the base of ‘growth-and-change’ processes typical of any city in Southern Europe (Arbaci 2007; Maloutas 2007; Kandylis et al. 2012). In this way, economists, geographers, sociologists, and planners, proposed several interpretative models to describe the ‘Mediterranean city’ archetype (Di Feliciantonio and Salvati 2015). Following a long debate on the stereotypical interpretation of urban development in Southern Europe (Ciommi et al. 2018), scholars were progressively abandoning the ‘myth’ of a unifying Mediterranean city model (Giannakourou 2005; Catalán et al. 2008; Chorianopoulos et al. 2014). They are instead moving to more general reflections on urban change, focusing together on settlement models and socioeconomic development patterns (Vaughan and Arbaci, 2011; Gkartzios et al. 2017; Kazemzadeh-Zow et al. 2017). Diversification, entropy, fragmentation, isolation, and fractality became some of the most suitable notions interpreting contemporary urban models in Southern Europe (Zambon et al. 2018, 2019; Salvati et al. 2018; Rodríguez-Pose and Storper 2020).

For many decades, Mediterranean cities developed in quite informal ways (Ciommi et al. 2019). Far from reaching a consensus on the definition of these spatial practises (Arbaci 2019), urban informality can be unswervingly adopted to label deregulated (or weakly regulated) development and activities in public spaces through which residents produce the city itself (Lydon and Garcia, 2015; Devlin 2018; Harris 2018). Being often associated with a neoliberal approach to metropolitan governance (Mayer 2007), urban informality reflects the pressure stemming from contextual dynamics (income polarization, impoverishment of the middle class, decreasing social integration because of marginalization of minorities) characteristic of a given city (Cucca and Ranci 2016).

These aspects, in combination with a rising trend toward place-based competition approaches to foster regional development, have often led to spatial structures rooted on unequal distribution of local resources and low inclusiveness, with distinctive processes of class segregation, gentrification, and social filtering (Panori et al. 2019; Rodríguez-Pose and Storper 2020; Trounstine 2020). However, earlier studies agree on the fact that attributes reflecting social heterogeneity, landscape configurations, settlements, and local functions underlying long-term informal development reflect, only in part, the inherent complexity at the base of contemporary metropolitan transformations (Lara-Hernandez et al. 2020). To delineate recent changes in the socio-spatial structure across metropolitan regions, analysis of economic indicators with an evident social impact is therefore meaningful (Chorianopoulos et al. 2010; Gkartzios 2013; Rontos et al. 2016). In this vein, income disparities are assumed to reflect together class and job segregation, distinguishing local contexts with diverging socioeconomic profiles likely better than any other indicator derived from official statistics (Siatitsa et al. 2020).

Moreover, although income segregation can be assumed as neutral in essence (OECD 2018), it may nevertheless be highly dysfunctional and problematic in widening intra-urban inequalities (Balampanidis et al. 2021). On the one hand, the suggested scenario of informal urban patterns determining, for a long time, social stratification and economic polarization in Southern Europe, seems far from Northern and Western European urban models (Leontidou 1990; Burgel 2004; Maloutas 2004, 2007). On the other hand, Mediterranean cities still are representative examples of disordered urban growth, discontinuous settlement structures, uncoordinated local economies, and residential sprawl, also in recent times, despite more formalized and participated planning processes (Maloutas and Karadimitriou 2001; Arbaci and Tapada-Berteli 2012; Remoundou et al. 2016).

Assuming urbanization as a leverage of a new spatial (dis)order within (and between) metropolitan regions, Merrifield (2013) questioned “how shall we reclaim the shapeless, formless and boundless metropolis as a theoretical object and political object of the progressive struggle?” In this perspective, our study proposes a rethinking of the urban question starting from an empirical analysis of latent transformations in a specific class of world cities (i.e. large agglomerations in between the global ‘north’ and ‘south’ – at the periphery of the most advanced European countries). In these regards, Mediterranean cities have undergone rapid transformations along a particularly complex development cycle alternating sequential phases of expansion and decline (Salvati et al. 2016).

More specifically, this study provides a spatially explicit analysis of a basic socioeconomic indicator - the average (per-capita) declared income in Athens (Greece) - with the aim at depicting the recent evolution of a representative Mediterranean city. We compared results of an exploratory analysis based on data mining with bibliographic findings proposed in earlier studies on the same area, exploiting comparable definitions, indicators, spatial scales, and analysis’ techniques (Morelli et al. 2014). In line with the abovementioned concepts, municipalities were regarded as the elementary unit of analysis (Salvati and Serra 2016), whose findings are easily interpretable by policymakers and planners (Rontos et al. 2016), and can be discussed in light of the intrinsic impact of socioeconomic variables on urban development (Carlucci et al. 2020). Comparison with findings of earlier studies finally allow for a specific analysis of income and wealth dynamics during two phases of the recent cycle in Athens: urbanization (between the late 1960s and the early 1990s) and suburbanization (mid-1990s to early 2010s). We assumed that, in line with earlier studies (Di Feliciantonio and Salvati 2015; Di Feliciantonio et al. 2018; Salvati 2019), income inequalities may follow distinctive patterns during these two phases, the one basically oriented along the urban-to-rural gradient during urbanization, and the other associated to a more latent, peri-urban-to-urban gradient during suburbanization. The empirical results of our study provide an explicit verification of this assumption.

In a broader debate of urban science, the case for Athens is interesting for different reasons: first, as an example of the inherent transition of Southern European cities from compactness toward moderately polycentric spatial configurations – this, in turn, representing a more generalized challenge all over Europe (Pili et al. 2017); second, as a unique case of tumultuous metropolitan change fuelled by the 2004 Olympic Games (Morelli et al. 2014). The approach proposed here is based on multivariate exploratory statistics and non-parametric correlations investigating the relationship between income segregation and the local context, of interest for planning purposes (Arbaci and Malheiros 2010). Results of the analysis contribute to delineate (and interpret the inherent complexity of) changing metropolitan contexts, identifying latent linkages among socio-spatial structures, economic configurations, and settlement morphology.

### Methodology

#### Study area

**Fig. 1:**
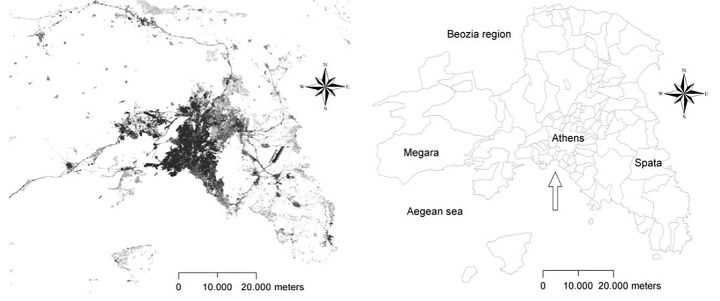
Athens’ urban footprint (left) and municipal boundaries representing the study area (right)

The present study focuses on the Athens’ metropolitan region encompassing the geographical boundaries of mainland Attica in Central Greece (Fig. 1). The area is partitioned in four prefectures (Athens, Piraeus, East Attica, and West Attica) administered by 115 municipalities before the national reform of local councils (the so called ‘Kallikratis’ law enforced in 2011, reducing the number of municipalities to less than 60 in the area). Athens’ municipality (38 km^2^) administered the city core with more than 600,000 resident inhabitants (Di Feliciantonio et al. 2018). According with United Nations definition of urban agglomerations (2018), Athens is among the world’s cities with 1 million inhabitants or more, and it hubs the main economic and commercial activities, government and transportation in Greece, in addition to more than 30% of the total country population (Morelli et al. 2014).

**Fig. 2:**
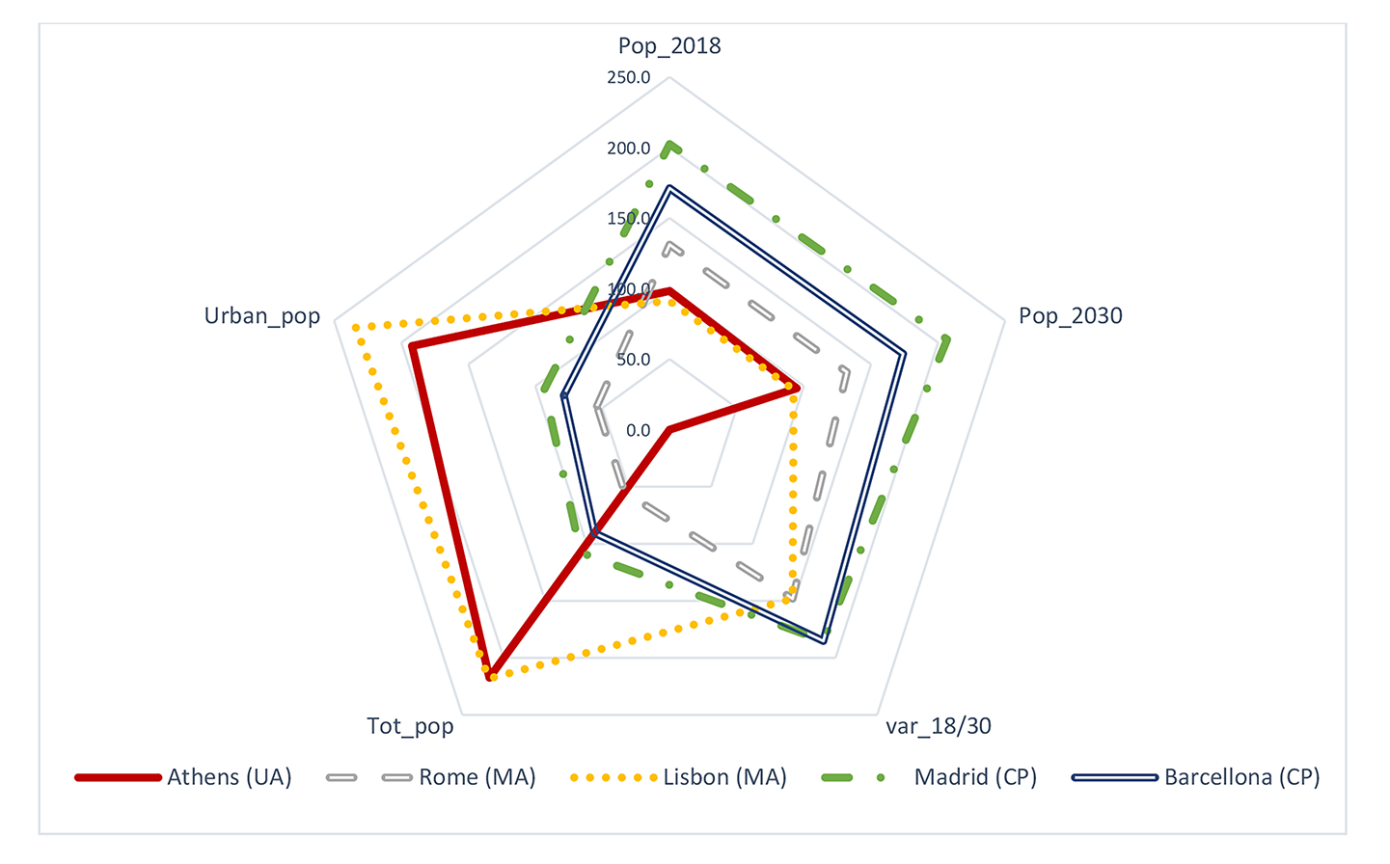
Demographic indexes for the largest cities in Southern Europe: total population, short-term population trend over time, and city population as a proportion of the country/area’s total or urban population in 2018 (per cent values). Indexes are computed with reference to mean values (= 100) of (large) cities in Southern Europe

Notes: Legend of radar-axis’ variables: (a) Pop_18: city population in 2018 (thousand inhabitants); (b) Pop_30: city population in 2030 (thousand inhabitants); (c) var_18/30: average annual rate of change (%) between 2018 and 2030; d) Tot_pop: city population as proportion of total regional population (%); e) Urban_pop: city population as proportion of total urban population (%). Country’s indexes are computed with regard to the mean values (= 100) of cities in Southern Europe. Geographical limits of a city were defined using the concepts of City Proper (CP), Urban Agglomerations (UA) and Metropolitan Area (MA). Source: own elaboration on United Nations (2018) data.

Compared with other major cities of Southern Europe (Fig. 2), population concentrated in a relatively small area (430 km^2^) known as the ‘Greater Athens’ area, hosting more than 7500 inhabitants/km^2^ (Rontos et al. 2016). While displaying intense demographic divides in urban and rural areas, the study area displays a substantially homogeneous compact settlement within the boundaries of the Greater Athens and more heterogeneous, less dense settlements on the fringe (on average, between 500 and 1000 inhabitants/km^2^). Although metropolitan growth has been particularly intense in the aftermath of World War I, the highest population density was observed in downtown Athens in the 1970s (> 15,000 inhabitants/km^2^). Early suburbanization consolidated dispersed settlements, especially - but not exclusively - along the sea coastline in the subsequent decades (Salvati and Serra 2016).

Rural districts have preserved traditional socioeconomic characters as far as landscape and population are concerned, while experiencing intense transformations following suburbanization in some cases (Cecchini et al. 2019). Growing as an industrial city up to the Second World War, Athens has later displayed an economic base oriented towards commerce, constructions, tourism, and public administration (Kourliouros 1997). The role of advanced services in regional economy grew substantially since the late 1980s (Chorianopoulos et al. 2010). Manufacturing and mining still represent economic sectors promoting development in some sub-central locations (Chorianopoulos et al. 2014). Economic stagnation and declining fertility are expected to translate into metropolitan shrinkage in a few years (Gounaridis et al. 2018; Panori et al. 2019; Carlucci et al. 2020).

Undulated morphology and mountains (Parnitha, Pendeli, Imitos, Egaleo) in Attica reduces buildable land and shapes the accessibility of some peripheral locations (Cecchini et al. 2019). Despite suburbanization before and after the 2004 Olympics, the Greater Athens’ area (Athens, Piraeus, and the surrounding municipalities) is still growing, as official statistics clearly show. Moreover, the intensity of soil sealing is higher downtown and lower in the suburbs, indicating the persistence of a mono-centric spatial organization (Pili et al. 2017).

#### Data and variables

The Athens’ socio-spatial structure was assessed considering the distribution of household wealth along the urban gradient through a basic indicator of (per-capita) average declared income by municipality derived from aggregated data released by the Greek Ministry of Finance. The spatial unit of analysis adopted in this study (municipalities) allows an appropriate investigation of changes in the spatial distribution of declared income along the urban gradient (Prodromidis 2012), and a complete integration of basic information from official statistics and field surveys (Panori et al. 2019). A total of 15 indicators referring to 2011 censuses or other official surveys were made available at the municipal scale from data released by Hellenic Statistic Authority, Ministry of the Environment, and Ministry of Finance: Municipal surface area (km^2^), Mean elevation (m), Proximity to the sea coast, Distance from Athens (km), Distance from Pireaus (km), Distance from Maroussi (km), Distance from Markopoulo Messoghias (km), Population density (inhabitants/km^2^), Population growth (%), Cropland (%), Forest land (%), Inhabitants *per* building, Residential buildings (%), Participation rate (%), and Class diversification (Pielou’s J index). These indicators were adopted and described extensively in earlier studies (Morelli et al. 2014; Rontos et al. 2016; Salvati and Serra 2016) with the final objective at profiling the socio-demographic and territorial context and defining the most significant gradients of income inequalities in Athens.

### Statistical analysis

The spatial distribution of contextual indicators was explored through maps based on the municipal boundaries released by Hellenic Statistical Authority. A correlation analysis using both parametric (Pearson) and non-parametric (Spearman and Kendall) coefficients – testing both linear and non-linear relationships – was carried out with the aim at assessing the pair-wise association between average (per-capita) declared income and the contextual indicators (illustrated above), testing at *p* < 0.05 after Bonferroni’s correction for multiple comparisons (Pili et al. 2017). Significant Pearson and Spearman (and/or Kendall) coefficients indicate a linear, pair-wise relationship between two variables (Gavalas et al. 2014). Significant Spearman (and/or Kendall) coefficients together with a non-significant Pearson coefficient indicate a non-linear relationship between a couple of variables (Morelli et al. 2014).

A Principal Component Analysis was carried out to identify the main factors and dimensions characterizing the socioeconomic profile of Attica’s municipalities (Rontos et al. 2016), reducing in turn the intrinsic redundancy of the multivariate input data matrix (Di Feliciantonio et al. 2018). Components with eigenvalues > 1 were extracted to produce two separate plots of loadings and scores (Di Feliciantonio and Salvati 2015). Component loading plot was analysed to identify multivariate, latent relationships among variables (Salvati 2014); score plot was analysed to infer significant geographical gradients from the distribution of elementary spatial units (municipalities) on the selected components (Salvati et al. 2018). Keiser-Meyer-Olkin and Bartlett statistics were run to evaluate the appropriateness of the factor model applied to the input data matrix, testing at *p* < 0.05 against the null hypothesis of inappropriate model’s formulation (Duvernoy et al. 2018). A magnification index was finally calculated as the ratio of the variance explained by the selected components in total input variance to the ratio of the number of components selected in the total number of components extracted (Cecchini et al. 2019). This index, ranging from 0 to ∞, evaluates the representation effectiveness of the selected components. Values < 1 indicate an ineffective representation of the data matrix, while values > > 1 reflect an optimal representation of the input variables.

## Results

**Fig. 3:**
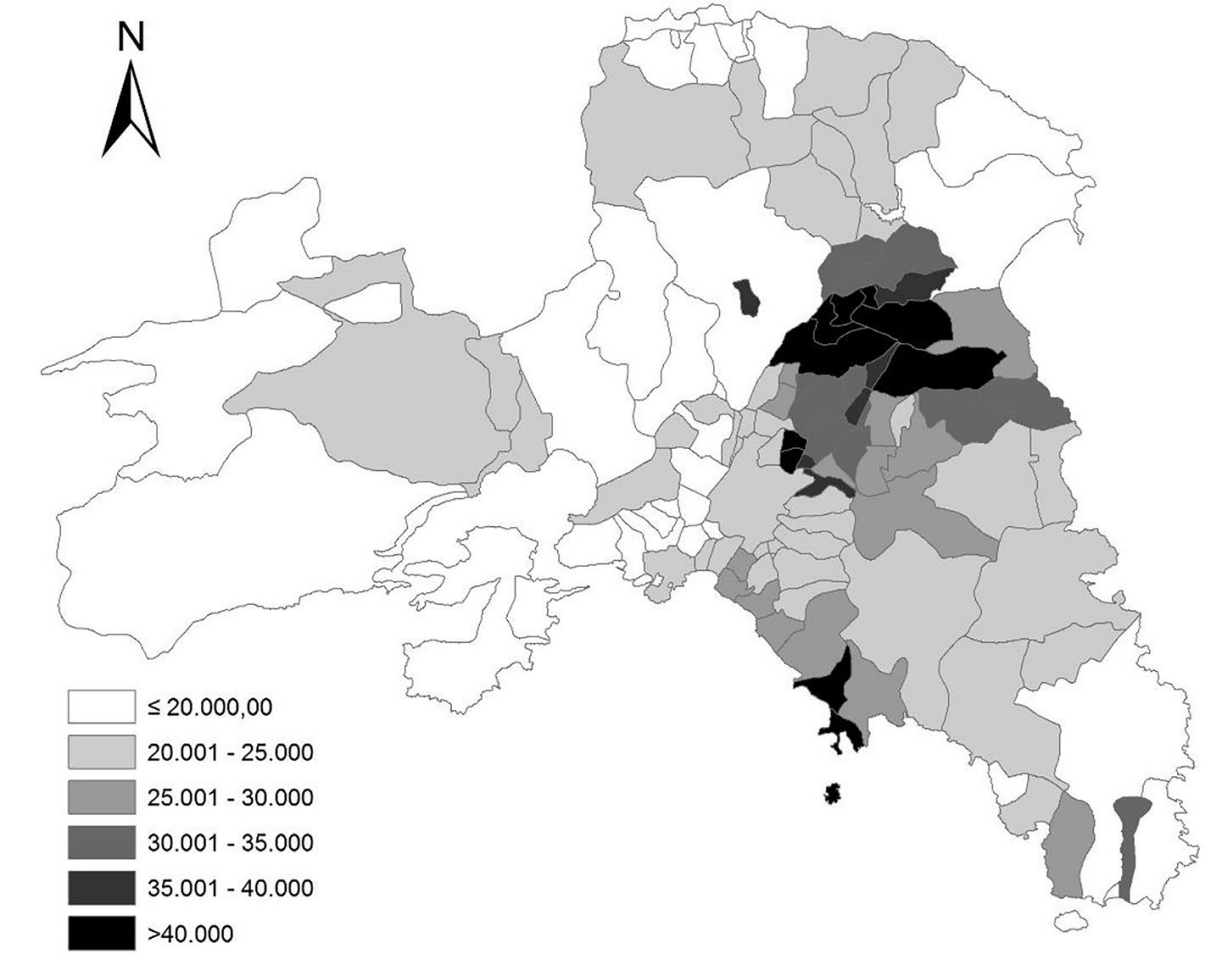
The spatial distribution of the average declared income (Euros per-capita) by municipality in the Athens’ metropolitan region (2011)

The analysis of declared incomes in Athens’ municipalities delineates a complex settlement configuration reflecting inherent inequalities (class/job/ethnic segregation, gentrification, and social filtering) along the urban gradient (Fig. 3). Considering different analysis’ scales, specific patterns were identified as follows: (i) a polarization in urban (affluent-moderately affluent) and rural (disadvantaged-moderately disadvantaged) municipalities (regional scale), (ii) an East-West divide reflecting a gradient from affluent to disadvantaged communities (metropolitan scale), and (iii) a mostly heterogeneous pattern mixing wealthy and economically depressed neighborhoods within small areas, in both central and peripheral locations (local scale).

**Fig. 4:**
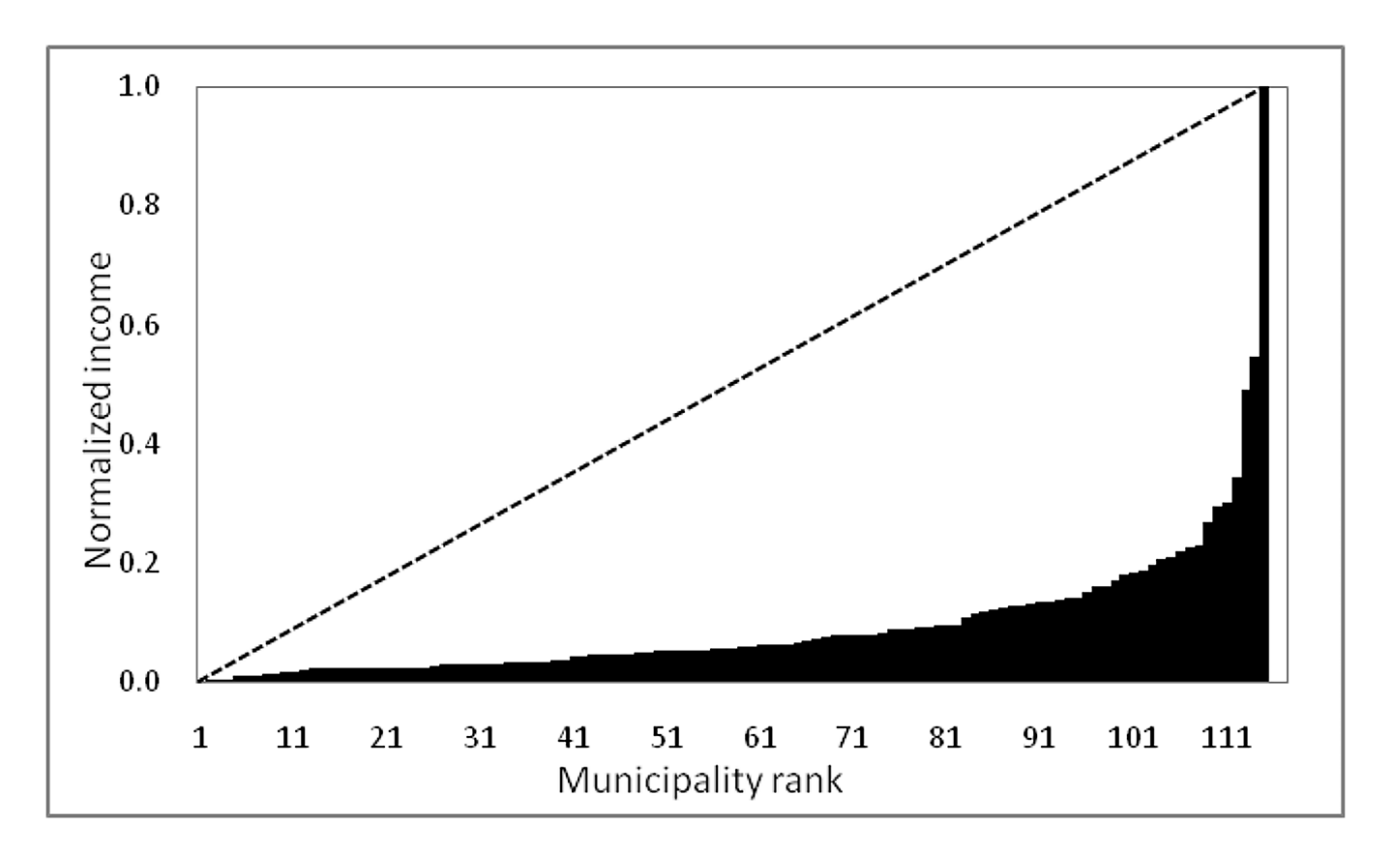
A concentration index for the distribution of (per-capita) declared income in Athens (municipal rank on x-axis and normalized income on y-axis)

Additionally, a specific cluster of municipalities with systematically higher income than the regional average was identified in the Northern district of Athens. This configuration consolidated after the Olympic Games thanks to infrastructural development, urban sprawl, and the progressive concentration of advanced services North of Athens. Figure 4 illustrates the statistical distribution of average incomes per municipality. Nearly 100 out of 115 municipalities had an average (per-capita) standardized income below 0.2 (in a scale between 0 and 1), suggesting the presence of intense wealth disparities in the area.

Pair-wise parametric and non-parametric correlation coefficients between average (per-capita) declared income and contextual indicators illustrated above were reported in Table 1. Results outline the importance of geographical location (accessibility), population density (concentration), and job market attributes in shaping the local distribution of income and wealth. More specifically, per-capita income decreased with the distance from the Central Business District of Athens consolidating around the Olympic Stadium in the affluent neighbourhoods of Maroussi and Kifissià, and with the distance from the International Airport. Pearson and Spearman coefficients were both significant, and indicate pair-wise linear correlations between the investigated variables. Average income increased with population growth and job participation rates, as well as with a measure of class diversification (Pielou evenness index). Significant Spearman and Kendall coefficients and a non-significant Pearson coefficient documented a non-linear relationship between average income and these contextual variables. Finally, average income increased moderately in residential contexts, outlining a linear relationship with the per cent share of residential buildings in total buildings *per* municipality.

**Table 1:** Parametric (Pearson) and non-parametric (Spearman and Kendall) correlation coefficients between (per capita, average) declared income and contextual indicators in Athens (significant coefficients at *p* < 0.05 were shown after Bonferroni’s correction for multiple comparisons)*

Indicator	Pearson	Spearman	Kendall
Distance from the Olympic Stadium, Maroussi (km)	-0.34	-0.53	-0.38
Distance from ‘E.Venizelos’ international airport (km)	-0.25	-0.53	-0.36
Population growth rate (2001–2011, %)		0.37	0.25
Share of residential buildings in total buildings (%)	0.33	0.28	
Job participation rate (%)		0.36	0.25
Class diversification (Pielou J entropy index)		0.44	0.31

* municipal area (km^2^), mean elevation (m), distance from downtown Athens and from Piraeus harbour (km), population density (inhabitants/km^2^), inhabitants per building, and per cent shares of cropland and forests in total landscape were not correlated with (per-capita, average) declared income

**Fig. 5:**
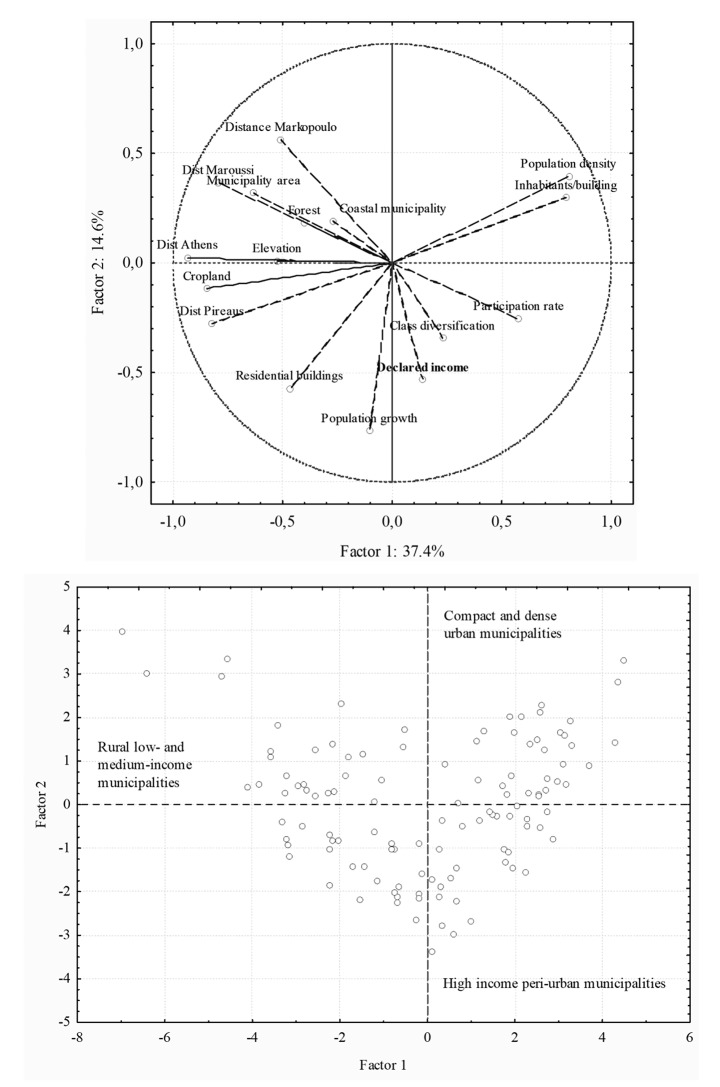
Results of a Principal Component Analysis applied to a data matrix of contextual variables and municipalities in the study area (upper panel: loading plot, lower panel: score plot)

The empirical results of a Principal Component Analysis (PCA) carried out on the same input matrix were illustrated in Fig. 5. The first two components extracted more than 52% of the total matrix variance and associated all input variables with significant loadings, providing a satisfactory representation of the data, in accordance with results of Keiser-Meyer-Olkin and Bartlett tests (both *p* < 0.001). Significant statistics indicate the appropriateness of the factor model applied to the input data matrix. A particularly high magnification index (3.9) demonstrates an optimal representation of the inputs along the first two axes. Component 1 (37.4% of total variance) summarizes the spatial distribution of declared income, population growth, and settlement characteristics along the urban gradient. Component 2 (14.6% of total variance) illustrates the land-use structure in the study area, opposing croplands and forests to population concentration and residential settlements. A more generalized interpretation of these findings delineates two important dimensions in the spatial organization of the study area. Component 1 illustrates how social diversification, personal income, and population expansion decreased with the distance from inner city, i.e. along the urban-rural gradient. Component 2 basically reflects economic disparities at the local scale, evidencing agglomeration factors at the base of urban growth and a more subtle urban-suburban gradient

## Discussion

To understand intrinsic mechanisms and socioeconomic forces shaping regional disparities in peripheral urban agglomerations of Southern Europe, we assumed that average declared income is an indicator of changes in the socio-spatial structure and the evolving economic dynamics (Maloutas 2007). Based on a comprehensive analysis of the spatial distribution of per-capita declared income in Athens’ municipalities and an extensive literature review, our study provides an original interpretation of income inequalities and class segregation in a representative Mediterranean city.

Morphology and spatial organisation of Athens’ agglomeration both reflect the intrinsic impact of multiple factors fuelling economic transformations and social change (Couch et al. 2007), evidencing a key role of demographic aspects, class segregation, land tenure, and political issues, as already observed in other Mediterranean cities, such as Rome, Barcelona, and Toulouse (Salvati 2014; Serra et al. 2014; Duvernoy et al. 2018). The interplay of different classes in the ‘metropolitan arena’ produced a particularly heterogeneous socio-spatial structure based on independent micro-entities and mixed ‘island’ settlements with characteristic class segregation (Maloutas 2007; Tsilimigkas et al. 2016; Panori et al. 2019), shaping social inequalities at wider scales (Kandylis et al. 2012). A comparison of the empirical results of our analysis with earlier studies indicates how the spatial structure of the city did not changed markedly in the last 30–40 years. More specifically, earlier studies focusing on income inequalities in the 1970s (Burgel 2004) and the 1980s (Leontidou 1990), reveal substantially similar socioeconomic divides at both the regional and local scale in Athens (Vradis 2014). Spatial planning oriented toward delocalization of the industrial areas since the 1970s (Kourliouros 1997) influenced only weakly the spatial configuration of the economic activities on a regional scale (Beriatos and Gospodini 2004).

Despite minor changes in the Athens’ urban structure mainly due to Olympic Games (Gospodini 2009), declared incomes showed a strongly polarized distribution on a municipal scale, which is comparable to what was observed in the past (Leontidou 1990; Burgel 2004; Maloutas 2004). Multivariate analysis highlights the spatial linkage between class segregation, residential settlements, population growth, and declared income (e.g. Gavalas et al. 2014). Results of the analysis depict a heterogeneous social geography on a regional scale based on three settlement ideal-types (urban, suburban, rural: see components’ scores in Fig. 5).

Two geographical gradients were identified as important factors shaping income inequalities in Athens. The former contrasts medium-high income (urban) municipalities with medium-low income (rural) municipalities. The latter was found on local (urban) scale, and opposes hyper-compact (urban) neighbourhoods to semi-dense, residential (suburban) settlements (Leontidou 1996a). Polarized socio-spatial contexts within the same metropolitan area reflects the differential impact of urban growth on the territorial configuration of settlements and economic activities (Arapoglou and Sayas 2009; Gounaridis et al. 2018; Panori et al. 2019).

**Fig. 6:**
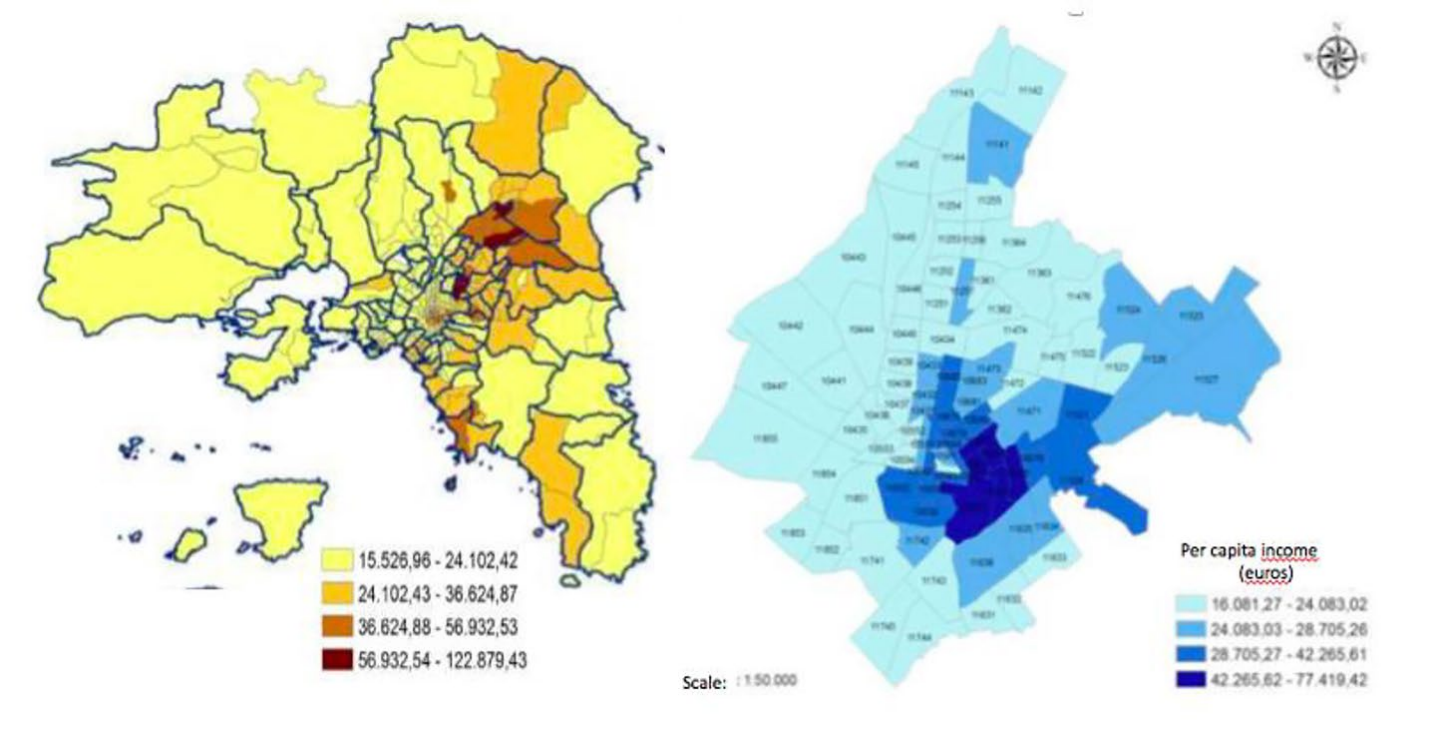
Spatial distribution of average, per-capita income (2011, Euros) in the Athens’ metropolitan region by post code unit (left) and a detail of downtown Athens was provided at the same spatial resolution (right)

The results of a micro-scale analysis of per-capita income by post-code in downtown Athens (Fig. 6) confirm the importance of the East-West (regional) gradient of income inequalities and class segregation, paralleling an intense polarization in affluent and depressed communities at the local scale (Morelli et al. 2014). In line with the working hypothesis of this study, the shift from a socio-spatial polarization in high-income central districts and low-income rural areas - typical of urbanization - to a more evident divide in medium-low income urban neighbourhoods and medium-high income peri-urban areas - characteristic of suburbanization – reflect the stratification of different social forces in Athens (Couch et al. 2007). These dynamics were already observed in other Mediterranean regions (Degen and García 2012; Kandylis et al. 2012; Salvati 2014; López-Gay and Salvati 2021).

Based on these transformations, the current Athens’ structure reflects a dualistic spatial pattern of income inequalities (urban vs. rural and suburban vs. urban divides), making the city as one of the most interesting examples of diversified forms and functions characteristic of the Mediterranean urban agglomerations (Carlucci et al. 2017; Zambon et al. 2017; Salvati et al. 2018). Governmental measures of industrial delocalization (Kourliouros 1997) and the intrinsic transformations in the 2004 Olympics (Beriatos and Gospodini 2004), have progressively replaced settlement informality and planning deregulation in Athens (Salvati, 2016), revealing the importance of policies mitigating the negative impact of social segregation and adapting to divergent urban contexts (Leontidou 1996b; Malheiros 2002; Sampson et al. 2017).

A thorough understanding of socio-spatial mechanisms underlying urban change definitely contributes to clarify settlement, land-use and economic dynamics (e.g. Filandri and Olagnero 2014). Such findings may call for the adoption of renewed policy tools that contribute to decision-making processes tuned finely with the increased complexity of contemporary cities (Zambon et al. 2019). Our study documents the relevance of a socioeconomic indicator (per-capita income) derived from computation on aggregate tax declarations’ data, to delineate (i) socio-spatial disparities, (ii) the importance of urban-rural (or urban-suburban) geographical gradients in wealth unbalances at the metropolitan scale, and (iii) the appropriateness of a multi-scale analysis of income inequalities, from regional to local levels. These findings contribute to develop a more general framework to understand differences in long-term trajectories of urban systems in line with earlier assumptions by Kloosterman and Lambregts (2007), i.e. suggesting a deeper investigation of two latent dimensions of growth, namely the level of capital accumulation and the level of capital concentration. The data mining approach adopted in our study can be particularly useful when integrating a refined analysis of capital accumulation and wealth with a local-scale investigation of socio-spatial disparities.

## Conclusions

The present study demonstrates how declared incomes were found correlated not only with the economic dynamics and performances of local districts, but also with the local socio-demographic context. These results allow for a spatially explicit assessment of social dynamics at the base of income inequalities. Analysis of declared incomes is particularly appropriate for the study of socioeconomic disparities at a disaggregated geographical scale, namely municipalities and/or local districts. Based on these premises, per-capita income derived from tax returns’ records was demonstrated to be a proxy of urban change and metropolitan dynamics. Investigation of the evolving spatial structure typical of modern cities contributes to inform policies for a more sustainable urban growth. Results of such investigations in turn suggest how a diachronic analysis of statistical indicators may shed further light in the debate about the changing spatial organization of metropolitan regions. The empirical findings of our study finally give the appropriate relevance to specific administrative data sources, such as the fiscal declarations derived from individual records collected by national Ministries of Economics and/or Financial Agencies, thanks to their informative contribution to official statistics.

## Data Availability

Data and materials are available on request.
